# COVID-19 and substance use disorders: a review of international guidelines for frontline healthcare workers of addiction services

**DOI:** 10.1186/s12888-022-03804-7

**Published:** 2022-03-31

**Authors:** Edoardo G. Ostinelli, Katharine Smith, Caroline Zangani, Michael J. Ostacher, Anne R. Lingford-Hughes, James S. W. Hong, Orla Macdonald, Andrea Cipriani

**Affiliations:** 1 Department of Psychiatry, University of Oxford, Oxford, UK; 2 Oxford Precision Psychiatry Lab, NIHR Oxford Health Biomedical Research Centre, Oxford, UK; 3 Oxford Health NHS Foundation Trust, Warneford Hospital, Oxford, UK; 4 Department of Psychiatry and Behavioral Sciences, Stanford University School of Medicine, Stanford, CA USA; 5 Department of Psychiatry, Palo Alto Health Care System, Palo Alto, CA USA; 6 Division of Psychiatry, Department of Brain Sciences, Imperial College London, London, UK; 7 Central North West London NHS Foundation Trust, London, UK

**Keywords:** COVID-19, Substance use disorder, Guidelines, Evidence-base recommendations, Alcohol, Opioid substitution treatment, Needle and syringe programme

## Abstract

**Background:**

People with substance use disorders may be at a greater risk of contracting COVID-19 infection and developing medical complications. Several institutional and governmental health agencies across the world developed ad hoc guidance for substance use disorder services and care of individuals misusing substances. We aimed to synthesise the best available recommendations on management and care of people with or at risk of substance use disorders during the COVID-19 pandemic from existing guidelines published in UK, USA, Australia, Canada, New Zealand, and Singapore.

**Methods:**

We systematically searched existing guidelines and websites from 28 international institutions and governmental bodies in the context of the COVID-19 pandemic (May 4^th^ 2021). We summarized the extracted data as answers to specific clinical questions.

**Results:**

We organised the available recommendations from 19 sources in three sections. First, we focused on general advice and recommendations for people who misuse alcohol or drugs during the COVID-19 pandemic, the design of contingency plans, safeguarding issues for children and families of service users and advice to the public, patients, and carers. Then, we summarised specific guidelines for people who use illicit drugs and related services, such as opioid substitution treatment and needle and syringe programmes. Finally, we provided a synthesis on specific recommendations for services supporting people who misuse alcohol and key topics in the field, such as management of alcohol detoxification and safe transition between supervised and unsupervised consumption.

**Conclusions:**

Available guidance reflected different approaches, ranging from being extremely cautious in providing recommendations other than generic statements to proposing adaptation of previously available guidelines to confront the challenges of the COVID-19 pandemic. After the early phase, guidance focused on reduction of infection transmission and service delivery. Guidance did not provide advice on infection prevention via vaccination programmes and service access strategies tailored to individuals with substance use disorders.

**Supplementary Information:**

The online version contains supplementary material available at 10.1186/s12888-022-03804-7.

## Background

The worldwide prevalence of substance use disorders (SUD) is estimated to be around 100 million for alcohol use disorders and between 27–41 million for people who are opioid dependent [1, 2]. Drug and alcohol use disorders are ranked 16^th^ and 20^th^, respectively, as leading causes of global burden of disease in adults aged 25 to 49 years old [3]. The coronavirus disease 2019 (COVID-19) pandemic presents significant challenges for people with SUD, magnifying social and economic inequalities further, especially for groups at higher overall risk such as immigrants and ethnic minorities [4–7].

People with SUD may be more susceptible to developing COVID-19. They may have a higher burden of comorbid medical and mental health conditions, be less likely to be tested for severe acute respiratory syndrome coronavirus 2 (SARS-CoV2), and live in social conditions which make it difficult to comply with home isolation (e.g. homeless, living in shelters) [8–10]. People with SUD may also face increased barriers to accessing health services for substance misuse [4, 5]. In addition, treatment providers may struggle to maintain adequate levels of continuity of care while protecting both patients and healthcare workers from COVID-19 [5].

Governmental health agencies across the world have rapidly developed additional guidance on treatment services, with adaptations to national legislation and policies, to try and meet the specific needs of patients with SUD during the COVID-19 pandemic [5]. However, the landscape of evidence-based clinical guidance is fragmented, and it is not easy for mental health professionals to find the information they need in a timely fashion.

To aid the busy clinicians in rapidly accessing reliable sources of existing guidelines in this area, we systematically searched and synthesised the best available guidance of both governmental and non-governmental agencies in the UK, USA, Australia, Canada, New Zealand, and Singapore, focusing on treatment of SUD during the COVID-19 pandemic and its aftermath.

## Methods

A multidisciplinary team (including mental health clinicians, researchers, methodologists, and a pharmacist) within the Oxford Precision Psychiatry Lab [11] systematically searched English language websites of organisations 1) defined as governmental institutions, professional bodies, health technologies agencies, international agencies, and scientific societies, 2) either international or from a list of English-speaking countries (i.e. United Kingdom, the United States, Australia, New Zealand, Canada, and Singapore), and 3) publishing guidelines or guidance on the management of substance and alcohol misuse and SUD in the context of the current COVID-19 pandemic and afterwards. We defined “substance” and “alcohol” as any substance or alcohol with abuse potential or whose use might cause dependence, or it is usually considered of interest for services dedicated to addiction. We used an a priori defined approach (further details can be found online), [12] already validated in synthesising guidelines on several topics [13–15]. At least two researchers (EGO, JSWH, OM, KS, and CZ) searched independently across the following sources in English until May 4^th^ 2021:

- Governmental institutions: Public Health England (PHE), Centers for Disease Control and Prevention (CDC), US Department of Labor, Singapore Ministry of Health (SMH), Health Canada (Government department), Australian Government Department of Health, Substance Abuse and Mental Health Services Administration (SAMHSA), and Canadian Society of Addiction Medicine (CSAM).
- Professional bodies: Royal College of Psychiatrists (RCPsych), Royal College of Nursing (RCN), Royal College of Physicians (RCP), American Psychiatric Association (APA), Singapore Psychiatric Association (SPA), Singapore Medical Association (SMA), Canadian Psychiatric Association (CPA), Royal Australian and New Zealand College of Psychiatrists (RANZCP).
- Health Technology Agencies (HTA): The National Institute for Health and Care Excellence (NICE), Healthcare Improvement Scotland.
- International agencies: World Health Organization (WHO), Inter-Agency Standing Committee (IASC), UNICEF, European Monitoring Centre for Drugs and Drug Addiction (EMCDDA).
- Scientific societies: The National Association of Psychiatric Intensive Care and Low Secure Units (NAPICU), RCPsych with British Geriatric Society and European Delirium Association, Massachusetts General Hospital Psychiatry, World Psychiatry Association (WPA), British Association of Psychopharmacology (BAP), Scottish Health Action on Alcohol Problems (SHAAP).

Further sources were hand-searched from the references of each website. Given that summarising data from primary studies was beyond our scope, we did not search reference databases (e.g. PubMed, EMBASE). Nonetheless, should we come across a particularly relevant publication, either supporting or in disagreement with available recommendations, we included it to better outline the context of a specific question. A search on Google was also completed using keywords relevant to COVID-19 (e.g. COVID-19, coronavirus, SARS-CoV-2 [severe acute respiratory syndrome coronavirus 2]), substance and alcohol use disorders and related treatments (e.g. opioid, buprenorphine), and guidelines (e.g. guideline, guidance, recommendation). Queries or disagreements were resolved by discussion with a third researcher (AC), and the team collaborated with international experts in the field (ARLH, MJO) to keep the guidance global, focused, and comprehensive. We incorporated the feedback of clinicians and mental health professionals, and the final synthesis of guidelines was grouped in a questions and answers format for ease of consultation, with key sentences highlighted in bold. The recommendations were classified as “General advice and recommendations”, when applicable to the whole population potentially accessing healthcare services due to substance or alcohol misuse, or “specific guidelines” when tailored to distinct sub-groups of service users. Finally, we appraised the identified recommendations according to type of source organisations and institutions (i.e. governmental institutions, professional bodies, healthcare technology assessment (HTA), international agencies, scientific societies).

## Results

We collected recommendations from 19 sources, [16–34] of which the majority (*n* = 11, 58%%) were from governmental institutions (please check Additional file 1 for the Preferred Reporting Items for Systematic Reviews and Meta-Analyses (PRISMA) checklist). The other identified sources were international agencies (*n* = 3), healthcare technology agencies (*n* = 2), professional bodies (*n* = 2), and scientific societies (*n* = 1). The identified sources focused on promoting telepsychiatry in routine care, providing detailed recommendations on how to deliver digital assessments to abide by the social distancing requirements. Moreover, available guidance emphasised the potential benefits of digital services, such as an increased level of anonymity for consultancy services, though inevitably some services require face-to-face interaction. A blended or mixed approach was thus recommended, such as in-person assessments delivered for people with a moderate level of dependence. Where possible, an increased level of flexibility was prompted in the provision of take-home treatments.

The full guidance is reported in Table 1. Section 1 of the table is about general advice on issues such as particular risks for people who misuse alcohol or drugs during the COVID-19 pandemic (including those who are not on treatment), the design of contingency plans, safeguarding issues for children and families of service users and advice to the public, patients, and carers. Section 2 of the table provides specific advice for services supporting people who use illicit drugs, with an added focus also on the available recommendations for needle and syringe programmes. The third and final section summarises specific advice for services supporting people who misuse alcohol, the management of alcohol detoxification, Wernicke’s encephalopathy, and the co-morbidity of alcohol use disorder and other mental illness.

**Table 1:** Guidance and information governance on substance use disorder and misuse of alcohol and drug. We used colour coding to highlight the different sources of information (see bottom of the table for details). Legend. AUDIT = Alcohol Use Disorders Identification Test. CDC = Centers for Disease Control and prevention. CG = Clinical Guideline. CO2 = Carbon Dioxide. COPD = Chronic obstructive pulmonary disease. COVID-19 = Coronavirus virus disease 2019. IM = Intra-Muscular. NIAAA = National Institute on Alcohol Abuse and Alcoholism. NHS = National Health Service. NICE = National Institute for Health and Care Excellence. NSP = Needle and syringe programme. OST = Opioid substitution therapy. PHE = Public Health England. PPE = Personal Protective Equipment. RCPsych = Royal College of Psychiatry. SAMHSA = Substance Abuse and Mental Health Services Administration. SHAAP = Scottish Health Action on Alcohol Problems. UK = United Kingdom. USA = United States of America. Sources of evidence: GI = Governmental Institutions. HTA = Healthcare Technology Assessment. IA = International Agencies. PB = Professional Bodies. SS = Scientific Societies

Clinical question		Guidance
**1. General advice**		
1a. What are the risks for people who misuse alcohol or drugs during the COVID-19 pandemic?		Direct risk factors: • [16] People with a history of drug misuse may be at greater risk of poor outcomes from COVID-19 compared to the general population due to a **significantly higher prevalence of chronic respiratory diseases** (asthma and COPD).[PB] • [17] **Greater vulnerability to the effects of viral infection, including COVID-19,** because of reduced immunity from poor health, drug and alcohol misuse, or medication for other conditions.[GI] • [17] **Risk of exacerbation of breathing impairment from COVID-19** due to use of drugs such as, for instance, opioids, benzodiazepines and pregabalin, and alcohol. However, the impact of several substances on COVID-19 is, currently, unclear.[GI] • [18] **Use of opioids may result in decreased oxygen and increased CO2 levels** in the blood by reduced breathing rate.[GI] • [18] **Stimulants such as methamphetamine, cocaine, and amphetamine constrict the blood vessels**, which could contribute to lung and respiratory damage and pulmonary hypertension in people who use it.[GI] Indirect risk factors: • [19] The COVID-19 pandemic may lead to increased feelings of fear, anxiety and isolation that can translate into a higher risk of relapse, substance misuse, disengagement from treatment, or non-compliance with treatment regimens.[IA] • [16] **Access to traditionally traded street opioids may be impacted** by global restrictions on movement, leading to a possible acceleration of the use of emerging synthetic potent opioids (e.g. fentanyl and related analogues). If supply chains are disrupted, **acute withdrawal from a variety of street drugs is more likely**, as well as the use of products users are uncertain of.[PB] • [20, 35] Societal consequences of the COVID-19 pandemic may include **reduced income**, **being isolated at home**, and **inconsistent access to usual levels of alcohol use**, resulting in an increased number and frequency of patients presenting acute alcohol withdrawal at a time when services are least able to deal with them. The impact of these consequences may be even higher for those from immigrant or ethnic minorities backgrounds. These communities have more limited access to health care resources even prior to the COVID-19 pandemic, tend to be in more marginalised economic situations and are subject to explicit and implicit bias.[GI] • [21] **Risks of disruption in access to drug services, clean drug-using equipment, and vital medications** are likely to increase the overall infection and withdrawal risks for people who misuse alcohol and drugs.[IA] • [21] **Sharing drug-using equipment between users** (e.g. cannabis joints, cigarettes, vaping or inhalation devices or drug paraphernalia) may increase the risk of infection.[IA] • [21] Use of illicit drugs may take place in **groups or in crowded settings**, thus increasing the risk of exposure to COVID-19.[IA] • [16] During the COVID-19 pandemic, hospitals, clinics, and ancillary health systems could be pushed to their capacity, and people with addiction – who are already stigmatized and underserved by the healthcare system – could experience even **greater barriers to treatment for COVID-19**.[PB]
1b. What should be considered when designing contingency plans during the COVID-19 pandemic?		It is important to ensure that the service users [19]:[IA] • **Continue to take medication as prescribed, particularly if they receive treatment with opioid medicines such as methadone or buprenorphine**. Maintaining access to opioid substitution treatment and to injecting equipment is paramount. • **Have a way of obtaining a regular supply of the medication**. • **Continue psychological support / support groups** during the pandemic, possibly delivered remotely or, if not feasible, at least with appropriate social distancing in person. **UK** When designing contingency plans**, prepare for a possibly higher number of episodes of withdrawal syndrome** during the periods of self isolation. Contingency plans should address the following issues [17]:[GI] • **Reduced or interrupted supply of medicines**, or access to them when pharmacies are closed. • **Supervised consumption may not be available** within a reasonable distance of some patients for whom it is clinically appropriate, due to pharmacy closure or restrictions on hours. Consideration should then be given to mitigations that reduce risk, such as **hostel staff holding medicines, pharmacy delivery of medicines if available, or lock boxes**. • **Reduced access to**, or interrupted supply of, **illicit drugs or alcohol**, **resulting in increased demand on services**. • **The possibility of a greater vulnerability to the effects of COVID-19** because of reduced immunity from poor health, drug and alcohol misuse, or medication for other conditions (this is possible in theory, but there is currently no empirical evidence to support increased vulnerability to COVID-19 in this client group). • **Risk of exacerbation of breathing impairment from COVID-19** due to use of drugs such as opioids, benzodiazepines and pregabalin, and alcohol. • **Increased risk of domestic abuse and violence** as people are forced to spend more time or self-isolate in the house and are unable to obtain drugs and alcohol. • **Increased risk of domestic violence and harm to children** whose parents or carers misuse drugs or alcohol, due to increased time together if children are not at school. Please also refer to the OxPPL synthesis of available guidance on domestic **violence and abuse during the COVID-19 pandemic**. Responses should include **ensuring that sufficient, rapid-access treatment capacity is available** (e.g. if people look for withdrawal support or substitute prescribing). PHE recommended to reintroduce a full range of support with both digital and face to face services (where possible), with a special focus of not widening inequalities further (e.g. access to digital therapies) [16]. Please refer to the OxPPL synthesis of available **guidance on digital mental health and telepsychiatry for further details**.[GI] In the UK, national guidance on clinical management of drug misuse and dependence and NICE guidance on harmful drinking and alcohol dependence should be used when considering these contingency plans.[HTA] • [17] Providers of drug and alcohol treatment services should liaise with local hospitals to ensure they are aware that **the symptoms of COVID-19 may be confused with withdrawal symptoms in a dependent drug or alcohol user**.[GI] • [17] Patients who are admitted to hospital (for any reason) and who show symptoms that could be attributed either to alcohol/drug withdrawal or COVID-19 should be **managed as if they have COVID-19 unless and until the test results show otherwise**, in addition to relevant ongoing treatment for withdrawal. Management will need to consider that treatment of withdrawal involves drugs such as benzodiazepines which depress respiration, please refer to the OxPPL available synthesis of **guidance on benzodiazepines and z drugs for further details**.[GI] **USA** Because of the substantial risk of coronavirus spread with congregation of individuals in a limited space such as in an inpatient or residential facility, admission to inpatient versus outpatient services should be carefully considered. SAMHSA has advised [22] that: • **outpatient treatment options**, when clinically appropriate, **should be used to the greatest extent possible**.[GI] • **inpatient facilities and residential programs should be reserved for those for whom outpatient measures are not considered an adequate clinical option**, i.e. for those with mental disorders that are life threatening (e.g. persons with life threatening substance use disorders, at high risk for overdose, complications from withdrawal).[GI] **Telehealth and telephonic services** can be used to provide evaluation and treatment of patients, as well as to implement individual or group therapies [22]. Please refer to the OxPPL synthesis of available guidance on digital mental health and telepsychiatry for further details.[GI]
1c. What safeguarding issues should be considered for children and families of service users?		**UK** [17] PHE has recommended to follow **local protocols and guidelines** in conjunction with the “children and families” section of the current guidance.[GI] **Referrals to children’s social care services or adult safeguarding services need to be made if a child or vulnerable adult is at risk of neglect or abuse** to properly assess the child’s needs and offer appropriate emotional and practical support.[GI] If staff of services for people who use drugs or alcohol discover a client is living with children or see that a client with children is now struggling to cope, they should **consider whether the family would benefit from further support from available services, community food banks and other resources**.[GI] **Safe storage boxes should be considered** (but bearing in mind that boxes have limited capacity that may not be enough for liquid medicines if take-home doses have been increased).[GI] The lockdown and the social distancing requirements **should not hinder the assessment of potential domestic abuse,** given that it can be assessed either through telephone or videocall. Please refer to the OxPPL synthesis of available **guidance on digital mental health and telepsychiatry** and on **domestic violence and abuse during the COVID-19 pandemic** for further details.
1d. What about the people who misuse drugs and alcohol but are not on treatment?		People who misuse drugs and alcohol and are not receiving drug and alcohol treatment may also be at greater risk than others in the community from COVID-19, and even more affected by changes in the supply of drugs and alcohol. **UK** [17] **If it can be supported, fast access to drug and alcohol treatment for these people will be important**. It may also be necessary to consider the nature and requirements of drug and alcohol treatment, with expectations of engagement and change reduced so that people are more willing to attend, at least for the duration of the COVID-19 pandemic. Satisfactory engagement into a treatment can be achieved also when delivered online – also remembering **the advantages that telepsychiatry can offer** (refer to the OxPPL synthesis of available **guidance on digital mental health and telepsychiatry** for further details).[GI] The supply of naloxone to those liable to use opioids, and of injecting equipment to those who inject drugs, **should continue to be made available as per national and local policies** (e.g. clients need to be risk assessed and shown how to use it).[GI] Consider referring people with substance use disorders not on treatment to **available online resources of support** (Sect. 1e).[GI]
1e. Where can the public, patients and carers get advice on problems with alcohol or drugs?		The current situation may limit the access to usual support networks. General principles for support also apply (these are suggested strategies for alcohol use disorders but are equally relevant for drug use disorders as well) [23]:[IA] • **Online interventions by professionals and mutual help groups can be less stigmatizing** as they offer greater anonymity and privacy.[IA] • **Advise patients to create a buddy and self-support system with someone trusted**, and to **reach out for extra help if needed**, such as online counselling, interventions, and support groups.[IA] • **Endorse physical distancing but avoid social isolation**: support the patient to call, text and/or write to friends, colleagues, neighbours, and relatives.[IA] • Advise patients to **maintain their daily routine as much as possible**, e.g. a daily workout, hobbies or mind relaxation techniques.[IA] **Telematic mutual support is available from several organisations and networks**: **UK** [24] • For people who have a substance use disorder:[GI] o Alcoholics Anonymous: https://www.alcoholics-anonymous.org.uk o Cocaine Anonymous: https://ca.org/meetings/united-kingdom/ o Drinkline: a free, confidential helpline for people who are concerned about their drinking, or someone else's. Call 0300 123 1110 (weekdays 9am–8 pm, weekends 11am–4 pm) o Marijuana Anonymous: http://www.marijuana-anonymous.org o Narcotics Anonymous: https://webdata.na.org/events/ o Reddit Recovery: https://www.reddit.com/r/REDDITORSINRECOVERY/ o Soberistas: https://soberistas.com/ o The Wales Drug & Alcohol Helpline (DAN 24/7): http://dan247.org.uk • For Families, Friends, and Significant Others:[GI] o Al-Anon/Alateen: https://www.al-anonuk.org.uk/ o Co-Anon: https://www.co-anon.org.uk o Families Anonymous: http://famanon.org.uk o The National Association for Children of Alcoholics: http://www.nacoa.org.uk/ o Nar-Anon: https://www.nar-anon.org/virtual-meetings **USA** [25] [26, 27] The NIAAA Alcohol Treatment Navigator can help finding telehealth alcohol treatment. It is possible to create a “**telehealth care team**” by combining a therapist with a licensed prescriber for medication support. It also provides links to mutual support groups for long-term recovery support.[GI] • For people who have a substance use disorder:[GI] o Alcoholics Anonymous: http://aa.org o CBT4CBT: an interactive cognitive-behavioural therapy program that uses videos and exercises to teach seven skills to help people cut down or quit drinking, https://cbt4cbt.com/providers/ o CheckUp & Choices: a digital self-help program that can help people to build the motivation and skills to change their drinking, https://checkupandchoices.com/alcohol/?utm_source=NIH&utm_medium=referral&utm_content=helpful-links-cnc-name o Cocaine Anonymous: http://www.ca.org o Crystal Meth Anonymous: http://www.crystalmeth.org o In The Rooms: https://www.intherooms.com/home/ o LifeRing: https://www.lifering.org/online-meetings o Marijuana Anonymous: http://www.marijuana-anonymous.org o Narcotics Anonymous: http://www.na.org o National Institute on Alcohol Abuse and Alcoholism – Alcohol Treatment Navigator: https://alcoholtreatment.niaaa.nih.gov o Reddit Recovery: https://www.reddit.com/r/REDDITORSINRECOVERY/ o Refuge Recovery: http://bit.ly/refuge-recovery1 o Secular Organizations for Sobriety/Save Our Selves: http://www.sossobriety.org o ShatterProof: https://www.shatterproof.org o SMART Recovery: http://www.smartrecovery.org o SoberCity: https://www.soberocity.com/ o Sobergrid: https://www.sobergrid.com/ o Soberistas: women-only international online recovery community, https://soberistas.com/ o Sober Recovery: https://www.soberrecovery.com/forums/ o Unity Recover + WEconnect + Alano Club: https://unityrecovery.org/digital-recovery-meetings o We Connect Recovery: https://www.weconnectrecovery.com/free-online-support-meetings o Women for Sobriety: http://www.womenforsobriety.org • For Families, Friends, and Significant Others:[GI] o Al-Anon/Alateen: http://www.al-anon.alateen.org o Co-Anon: http://www.co-anon.org o Families Anonymous: http://www.familiesanonymous.org o Nar-Anon: http://nar-anon.org o Unity Recover + WEconnect + Alano Club: https://unityrecovery.org/digital-recovery-meetings
**2. Specific advice for people who use illicit drugs**		
2a. Are there any special recommendations for people who use illicit drugs?		**UK** [17] **Considering access to opioid substitution treatment (OST),** treatment providers, pharmacies, commissioners, and Local Pharmaceutical Committees should be as flexible as possible, within the legal framework, to support safe delivery of OST:[GI] 1. Except for patients who are self-isolating, shielding or in areas where the availability of supervised consumption at pharmacies is severely limited, **clinical decisions regarding pick-up and supervision requirements should be now made in broadly the same manner as before the pandemic** and in line with the clinical guidelines.[GI] 2. **Prescribing arrangements for all patients on OST should be reviewed as normal**, and future arrangements should be based on repeat individual assessments.[GI] 3. In the initial phase of the response to the COVID-19 pandemic, many people were switched from supervised to unsupervised consumption of OST. For these patients, current assessments should include **how they responded to any relaxation in pick-up and supervision requirements**, **confirmation (not just self-report) that they are not using on top**, and **visual evidence that they are thriving**.[GI] 4. People who are clinically extremely vulnerable or have been advised to self-isolate (but not treated in hospital) should be asked to **nominate an individual to collect the dispensed medicine on their behalf**.[GI] • The nominated individual will **usually need the written instruction of the patient** (see a template letter to enable a representative to collect medicines).[GI] • If the patient cannot nominate someone, **a staff member may, with agreed authorisation, be able to collect and deliver the medicines**.[GI] • **Delivery can also be requested** from the pharmacy.[GI] 5. Consideration might be given to implementing a system whereby a small group of nominated individuals are authorised to collect medicines on patients’ behalf, if it can be done safely.[GI] 6. [28, 29] Supervised consumption is not a legal requirement under the 2001 Regulations. Nevertheless, when supervised consumption is directed on the prescription, any deviation from the prescriber’s intended method of supply should be documented and the justification for this recorded. Any such decision should be made in the best interests of the patient, ideally always involving the prescriber. Remember also that new scripts are not required if the community pharmacy has to stop supervising the consumption of the prescription.[HTA, SS] Considerations should also be given in terms of **how to start or continue treatments and clinical management during the COVID-19 pandemic**.[GI] 1. Detoxifications and dose reductions may have been deferred, with people encouraged to maintain stability during the initial period of uncertainty, but it may now be time to review these changes.[GI] 2. If unsupervised dosing is needed from the outset for people newly assessed for treatment, then there are safety advantages to buprenorphine compared to methadone. Patients with good support and stable circumstances can be provided with a multi-day supply of buprenorphine. Patients being initiated on to methadone treatment should generally collect their medicine daily from the pharmacy, followed by take-home doses when appropriate.[GI] 3. People restarting OST who were taking methadone in the recent past may be able to return to methadone after careful assessment but usually starting at a lower dose, titrated up again and with daily pick-up to start if available.[GI] 4. If only remote assessments are possible and drug testing is not possible, it may be possible to proceed with buprenorphine titration in known opioid-dependent patients as above, based on an adequate history.[GI] 5. The above approach is unlikely to be suitable for methadone, where drug testing will usually be needed unless there is a clear history of opioid use and tolerance in a known patient, with evidence that opioids have been used in the last 24 h.[GI] **Collaboration with and communication between services** is fundamental:[GI] • **Inform GPs of the changes in prescribing and amounts of OST medicines stored in homes where there are children, and inform local children’s social care services** if they are involved or if there are any concerns.[GI] • If providing care to released prisoners and detainees, work with health and justice to provide **rapid access to treatment** and to ensure safe **continuity of care**.[GI] • Work with police to **provide ****treatment for those taken into custody**.[GI] • Work with local services supporting isolation for **people who misuse drug and alcohol experiencing rough sleeping to ensure continuity of care**.[GI] **USA** [30] For settings where dedicated personnel are available to deliver OST medicines, such as in the USA, **SAMHSA has been recommended staff retreat a minimum of 6 feet to observe that the medications are picked up** by the patient or the designated person to receive the medications.[GI] Staff must ask the person who is retrieving the medication to identify themselves. Staff should determine that the person appearing to retrieve the medication is the patient or the household member named by the patient as having permission to do so.[GI]
2b. What are the available recommendations for needle and syringe programmes (NSPs)?		**UK** [17] PHE recommended that there is an adequate supply of injecting equipment:[GI] • **Increasing the amount of stock** held by needle and syringe programmes (NSP).[GI] • **Allowing service users to take more equipment** or providing packs with more equipment in them.[GI] • **More outreach and peer-to-peer supply** with appropriate social distancing.[GI] • **Allowing others to collect equipment** for someone or for general peer-to-peer distribution.[GI] • Considering other options such as **posting supplies**.[GI] Any changes in pharmacy-based NSPs will need to be agreed with the pharmacies involved.[GI] It may also be necessary, **as a last resort, to provide advice on cleaning injecting equipment**. More information on cleaning injecting equipment is in this video.[GI] **USA** [31] **CDC stated that NSPs should be considered by state, local, territorial, and tribal jurisdictions as essential public health infrastructure that should continue to operate during the COVID-19 pandemic.[GI]** During the COVID-19 pandemic, it is critical that NSPs have the capacity to ensure the safety of staff, volunteers, and clients. CDC provided a guidance that describes actions for jurisdictional public health authorities, as well as NSPs, to support the health and well-being of their staff and the clientele they serve (summarised below):[GI] • **Continue to provide sterile injection equipment and methods for disposal**, as well as skin cleansing supplies such as alcohol pads and hand sanitizer, to help reduce risk of COVID-19 infection, as well as to prevent skin and soft tissue infections related to unsafe injection practices.[GI] • **If necessary, change policies to increase the number of syringes each client can receive per visit to enable longer periods between visits**. This practice will minimize the need to access NSPs frequently and can help ensure sterile injection in the event of NSP closure or limited hours.[GI] o Ensure clients have adequate supplies to use sterile equipment with each injection. Dispense enough supplies to ensure continued ability of clients to inject with sterile equipment if NSP closure or limited hours may happen during the pandemic.[GI] • **Provide supplies through mobile services, delivery, or mail-order services**, whenever possible.[GI] • **Provide counseling services and referrals to care by telephone and/or using online tools**, where feasible.[GI] • Review existing service provision procedures to identify ways to minimize opportunities for COVID-19 exposure and transmission.[GI] • **Use or encourage others to use peer-based delivery models** (e.g., providing enough supplies to clients so they can distribute to other people who inject drugs who may be unwilling or unable to visit the program) to ensure sterile supplies are reaching people who need them most, even in the event of NSP closure.[GI] • If sterile injection equipment is unavailable or cannot otherwise be provided, **provide bleach, sterile water, and ****instructions for cleaning syringes**. This **does not ensure sterile injection**, but may reduce the risk of infectious disease transmission.[GI]
**3. Specific advice for people who misuse alcohol**		
3a. What advice can be given to people who misuse alcohol?		[23] People with an alcohol use disorder are at **greater risk of COVID-19**, not only because of the impact of alcohol on their health, but also because they are more likely to experience homelessness or incarceration than other members of the population. It is therefore essential, under the current conditions, that people who need help because of their alcohol use get all the support they need.[IA] • [23] **The present situation is a unique opportunity to quit drinking, or at least to cut down considerably**, as various social cues and peer pressure situations, such as parties, friends’ gatherings, restaurants and clubs, are (by necessity) avoidable.[IA] • [17, 32] However, **people dependent on alcohol should be advised not to just stop drinking alcohol**. **Reducing consumption gradually may also not be possible** for them as a cardinal feature of being dependent is ‘lack of control’ over their consumption (see Sect. 3b).[GI, PB] • [23] **Online interventions for alcohol use disorders by professionals and mutual help groups can be less stigmatizing** as they offer greater anonymity and privacy.[IA] • [23] **Advise patients to create a buddy and self-support system with someone trusted**, and to **reach out for extra help if needed**, such as online counselling, interventions, and support groups.[IA] • [23] **Endorse physical distancing but avoid social isolation**: support the patient to call, text and/or write to friends, colleagues, neighbours, and relatives.[IA] • [23] Advise patients to **maintain their daily routine as much as possible**, e.g. a daily workout, hobbies or mind relaxation techniques.[IA] Discuss with the service users and refute **common assumptions about alcohol and COVID-19** [23]:[IA] • **Consuming alcohol will not destroy the virus**, and its consumption is likely to increase the health risks if a person becomes infected with the virus. Alcohol (at a concentration of at least 60% by volume) works as a disinfectant on the skin, but it has no such effect within when ingested.[IA] • **Consumption of alcohol will not kill the virus in inhaled air**; it will not disinfect the mouth and throat, nor give protection against COVID-19.[IA] • Alcohol has a deleterious effect on the immune system and **will not stimulate immunity and virus resistance**.[IA] Advise people who misuse alcohol to and those with a potential at-risk use of alcohol to consult **one or more culturally appropriate resources **with useful information to recognise drinking levels and to help managing drinking, and. For instance, in the UK consider using resources from RCPsych, NHS UK, SHAAP, and Alcohol Change.
3b. How to manage alcohol detoxification?		**Remember that there are risks in abruptly reducing or stopping drinking in people who are severely alcohol dependent**. **Those who are at particularly high risk** of developing withdrawal complications and are more likely to require emergency medical treatment if they reduce or stop drinking abruptly include [17]:[GI] • Service users **drinking over 30 units of alcohol per day**.[GI] • Those who have **pre-existing epilepsy**.[GI] • Those who have a **history of seizures or delirium tremens during alcohol withdrawal**.[GI] For all presentations of alcohol withdrawal, assess [32]:[PB] • If they are **currently dependent or not** (e.g. AUDIT with a score > 20 and Severity of Alcohol Dependence Questionnaire Score > 15 can be used as an initial screen).[PB] • If they are withdrawing from alcohol and require medication.[PB] • The **risk of alcohol withdrawal** (i.e. previous seizures, delirium tremens, and medical history) and its complications.[PB] • The **risk to develop or of current Wernicke’s encephalopathy** based on history and clinical presentation (e.g. ataxia, confusion, ophthalmoplegia).[PB] [17] In the UK, during the initial response to the pandemic, alcohol detoxification may have been limited and conducted partly remotely. Where possible services should now return to delivering community detoxification or referring people to inpatient or residential detoxification in accordance with NICE guideline CG115.[GI] [17] If services are temporarily unable to offer community, inpatient or residential detoxification, competent staff should give **advice on alcohol harm reduction** (please refer to Sect. 3a).[GI] [17] PHE has published separate guidance to support dependent drinkers to cut down without medication. The advice is also relevant for family members and friends supporting the person.[GI] [17] Following clinical assessment, it might be appropriate to advise that the above defined **high-risk group continue drinking for the time being, preferably at a steady level** with no large alcohol binges or days without any alcohol, to avoid severe complications of withdrawal. They should do this **until it is possible to arrange appropriate medically supervised detoxification**.[GI] [17] Decisions about the **provision of community alcohol detoxification** should be made **on a case-by-case basis**, following clinical assessment. Suitability for community detoxification and risk assessment should be based on severity of dependence and complexity of additional needs, in accordance with NICE guideline CG115. People with moderate dependence, without additional needs or risk factors, will usually be suitable for community detoxification.[GI] **For service users with mild to moderate dependence** [17]**:**[GI] • Services should offer **face-to-face assessment and face-to-face monitoring at least every 2 days** during community detoxification wherever this can be carried out safely.[GI] • If not possible, a suitable detoxification regimen using a **prescription of a recommended benzodiazepine could be issued**, based on an assessment by a competent clinician. The patient would then be monitored **regularly through telephone conversations or video calls**.[GI] • The dose of benzodiazepine should be tailored to the level of severity of alcohol dependence as recommended by NICE guideline CG115.[GI] • Service users and carers should be warned of the **signs of severe alcohol withdrawal and advised to seek urgent medical care should they occur**.[GI] • Wherever possible, medication should be dispensed and delivered (or collected) every 2 days.[GI] • For service users living alone, community detoxification should only be offered in exceptional circumstances, following an assessment of relative risks and benefits. Consult the local policies on alcohol detoxification (see an example from the Oxford Health trust).[GI] **For service users with severe dependence or complex needs** [17]**:**[GI] • Those with severe dependence will usually require **residential or inpatient detoxification** and should generally be **advised to continue drinking steadily until they can access this**.[GI] • If they are unable to attend and they urgently need to stop drinking, they should receive a face-to-face assessment and should be monitored by a competent clinical team with involvement of senior clinicians.[GI] Anyone undertaking a detox should be **strongly encouraged and supported** to achieve a plan for relapse prevention prior to starting detoxification from alcohol [32]:[PB] o 1:1 or virtual group **psychosocial support/treatment**.[PB] o **Medication**: consult your local and national policies. For instance, in the UK the licensed medications include **acamprosate** and **naltrexone** as first-line treatments (NICE) followed by disulfiram due to potential for interactions. **Nalmefene** is a medication that can be given to alcohol dependent individuals to help them stop drinking alcohol if they do not immediately require alcohol detox (UK).[PB] • It may be appropriate to **give out 2–3 days of medication rather than the full detox regimen if uncertain whether individual will complete detox** or if there are concerns about how much medication they should have access to.[PB] [17, 33] When providing alcohol detox in any setting, be aware of the **risk of potentially fatal respiratory depression with benzodiazepines and opioids** and consider how it might relate to infection with COVID-19. **This caution in using benzodiazepines should be balanced against the risks of not adequately treating severe symptoms of acute alcohol withdrawal** (see the OxPPL synthesis of available guidance on benzodiazepines and Z-drugs for some general considerations on how to carefully weight their use during the COVID-19 pandemic).[GI] [17] In the UK, PHE suggested that during the restrictions of COVID-19 and subject to the availability of sufficient supplies, service users who are dependent on alcohol could be given **a one-month supply of thiamine on their first presentation at a treatment service**.[GI]
3c. Additional precautions for Wernicke’s encephalopathy		[32] Wernicke’s encephalopathy is a potential complication of excessive alcohol use.[PB] [32] Assessment of Wernicke’s encephalopathy **needs to be considered in all individuals undergoing alcohol withdrawal or detox** as it may develop due to the **increased metabolic load** on the brain during withdrawal, but **it may also develop if the thiamine intake reduces** (e.g. poor diet, not eating due to being unwell or in presence of infection).[PB] [32] Wernicke’s encephalopathy **is a medical emergency**, a high index of suspicion must be maintained.[PB] **If an individual is at risk of Wernicke’s encephalopathy** (e.g. missing meals, signs of peripheral neuropathy) [31]:[PB] • **Administer Pabrinex IM**, one pair of ampoules/day for 3 days (need not be consecutive but ideally within a week; consider if can be given prior to detox). The personnel involved in the administration should use PPE (for the UK, refer to the guidance about COVID-19 PPE by Public Health England.[PB] • **Pabrinex administration may rarely cause anaphylaxis. Patients given Pabrinex must be monitored for 30 min after administration and equipment for dealing with anaphylaxis should be readily available**.[PB] • Check if the individual is receiving ongoing oral supplementation. Whilst poor absorption of oral thiamine means it is not equivalent to Pabrinex for those clearly at risk, **consider role for oral thiamine supplementation in reducing risk longer-term**.[PB] • **Remain vigilant** for development of Wernicke’s encephalopathy.[PB] **If the individual shows signs of Wernicke’s encephalopathy** (e.g. ataxia, confusion, ophthalmoplegia) [31]:[PB] • **Refer him/her immediately to hospital** as per local protocol to receive parenteral thiamine.[PB]
3d. How do I manage individuals with co-morbid alcohol use disorder and other mental illness?		[34] Patients with alcohol use disorder are a vulnerable group due to the **high prevalence of co-morbid physical and mental health problems**, **both related to and independent** of alcohol.[HTA] Co-morbid alcohol dependence should **not be considered as a barrier to accessing secondary mental health community services** [34]:[HTA] • Ensure that patients with serious mental disorder and co-morbid alcohol dependence can access **admission and management as inpatients**, when needed.[HTA] • **Early identification on admission is essential to ensure appropriate management**, to avoid delay and expedite discharge, to minimise length of stay, and reduce likelihood of readmission.[HTA] • Endorse a **more integrated management of alcohol dependence to reduce crisis presentations** for patients managed in **community mental health services**.[HTA]

## Discussion

We have summarised the available guidelines published in English-speaking countries on several key topics relevant to the management of people with substance or alcohol use disorders and misuse of drugs or alcohol during the COVID-19 pandemic. Identified sources were primarily from governmental institutions. Available guidance reflected several changes to standard practice and care that occurred due to the impact of the COVID-19 pandemic on substance and alcohol use disorders treatment services. Key changes in recommendations focused on the role of telepsychiatry and increased flexibility in dispensing take-home treatments.

Identified guidelines widely promoted the use of digital consultations and telepsychiatry to maintain social distancing when delivering substance and alcohol use services whilst minimising perceived social isolation. The social distancing restrictions in several countries prompted a rapid implementation of telehealth and the dissemination of digital mental health practices at an unprecedented pace [13]. Telehealth services have the potential to overcome some limitations and restrictions of the traditional in-person approach [36]. For instance, digital peer-to-peer recovery support services allowed individuals to access 24/7 support, whilst leveraging the potential benefit of greater anonymity [37].

In the early phase of the COVID-19 pandemic, detoxification services needed to adapt the management and delivery processes of their activities. However, these changes to usual practice came with some drawbacks. Service disruptions and unmet support needs have been recorded as a direct result of the impact of the COVID-19 pandemic [36, 38, 39]. Such initial lack of adequate provision of care is not exclusive to the substance and alcohol misuse field, with early reports from mental health patients in China experiencing difficulties accessing general mental health services and support [40]. These instances highlight underlying inequities in health service access and gaps which are being worsened by the pandemic. Early reports suggested that young individuals and ethnic minorities could be amongst those facing greater challenges in benefitting from healthcare services [35, 38, 41]. Available guidance prompted services to prevent and address this issue, failing however to offer healthcare workers with tangible solutions or pragmatic action points.

In some cases, the initial adaptation of policies and recommendations to the COVID-19 pandemic significantly deviated from usual care. For instance, in the UK requirements for opioid substitution therapy prescription were relaxed. Drug testing was suspended as a requirement for buprenorphine prescription and, in the case of methadone, limited to individuals without a clear history of opioid use and tolerance and known patients with evidence that opioids have been used in the previous 24 h [17]. In-person attendance was formerly required for procedures such as urine drug screening to ensure safe treatment with methadone and buprenorphine. [42]. A potential risk of increased use of opioid substitution therapy cannot be excluded. Indeed, the most recent recommendations from PHE now advise the collection of confirmatory evidence of intake when assessing patients [16, 17].

As the pandemic has progressed, focus has shifted from immediate management and reduction of transmission to prevention via licensed COVID-19 vaccines. A key area of concern is vaccine hesitancy, in those with mental health difficulties more generally, [15] and specifically in those with SUD. Individuals with SUD have a greater risk of contracting a COVID-19 infection, [35] but are less likely to access preventative interventions such as vaccines [43]. Addressing equitable access to vaccines, as well as systemic and individual risk factors will be key to increasing uptake in this vulnerable group [44]. There is currently no specific formal guidance in addressing vaccine hesitancy in those with mental health difficulties or SUD. However, strategies aimed at increasing uptake in people with severe mental illness may be equally relevant in people with SUD. These include vaccination programmes within support services, alignment with other preventative health strategies including influenza vaccination, focused outreach, and monitoring uptake of vaccines [45].

Our work has some potential limitations. The search process was restricted to English language sources to synthesise available guidance from a list of English-speaking countries. The recommended resources in this paper are primarily limited to the UK and USA, whilst laws and policies regarding the treatment of and resources for people with SUD may differ between countries. For instance, this is indeed the case with regards to the treatment of opioid use disorder. Nonetheless, given the global nature of the COVID-19 pandemic, international collaborators have produced translations of our synthesis of guidance in several foreign languages and adapted it for local use [11, 13]. Since our aim was to synthesise guidelines and recommendations, our search strategy and data extraction processes were implicitly different in terms of replicability when compared to a systematic review of records from a database. To address this limitation, we adopted a comprehensive search and contacted experts in the field. We structured our work on the PRISMA checklist, adapting it where needed, to ensure the process was as systematic and rigorous as possible. For instance, results of our search strategy to locate additional potentially relevant international/national agencies are rapidly evolving and reflect the ongoing status of the COVID-19 pandemic. Nonetheless, our scope was to identify all the relevant governmental and non-governmental agencies, as opposed to systematic reviews relying on the number of records as the unit of analysis. We repeated a search for each country and checked all the links to other websites. We maximised transparency by listing all the identified sources searched and linking extracted information to the related website or document. Finally, we could not appraise the certainty of the evidence supporting the identified recommendations. The Grading of Recommendations, Assessment, Development and Evaluation (GRADE) system allows evaluation of structured guidelines with defined methodologies and their evidence. However, GRADE discourages any modification to the current approach [46] and its application was not possible due to the nature of our aims and how quickly organisations and institutions responded to the COVID-19 pandemic. New approaches are needed to reliably appraise the confidence in recommendations when time is essential (e.g. disasters and emergencies).

## Conclusions

Substance and alcohol misuse services had to rapidly and profoundly change to limit disruptions for service users and, where possible, continue the treatments previously in place. At the same time, the demand for services increased. More than 8 million adults in the UK are drinking at high risk, and a surge in the number of people addicted to opiates seeking help have been recorded [47]. Mental health care workers and researchers should use experience gained during the COVID-19 pandemic to address existing issues as well as to expand and improve the quality of the services offered. This will require a careful balance between the need to enhance flexibility while ensuring continuity of service delivery, and the need to maintain high standards of care [48]. When considering the available recommendations, clinicians should carefully weigh up potential risks and benefits on a case-by-case basis, especially when inconsistencies between newer recommendations and usual practice arise.

In the initial phase of the COVID-19 pandemic, regulatory agencies and professional bodies quickly provided guidance to address urgent issues. This rapid response was inherently based on limited evidence and resulted in several different approaches. Some sources of guidance were more conservative and limited to generic recommendations, whilst others suggested temporary amendments of pre-COVID-19 guidelines. Since then, a vast volume of scientific literature has been published on COVID-19, which present the opposite challenge of quickly synthesising available evidence. Future guidance should shift focus from acute restrictions to longer term management, such as the implementation of a comprehensive vaccination programme with equitable access and managing the mental health consequences of COVID-19. To do so, guidelines should source their recommendations from the growing body of literature by leveraging newly developed frameworks to provide up-to-date syntheses of the available evidence [49].

## Supplementary Information

**Additional file 1.**

**Additional file 2.**

## Data Availability

All data generated or analysed during this study are included in this published article.
