# Supplementary material for: COVID-19 and substance use disorders: a review of international guidelines for frontline healthcare workers of addiction services
Source: BMC Psychiatry. 2022 Mar 31;22:228. doi: 10.1186/s12888-022-03804-7 (PMC8968241; doi:10.1186/s12888-022-03804-7)
Supplement: Supplementary file 2 — Additional file 2. [file 12888_2022_3804_MOESM2_ESM.docx]

**List of organisations**

- Governmental institutions: Public Health England (PHE) (<https://www.gov.uk/government/organisations/public-health-england>), Centers for Disease Control and Prevention (CDC) (<https://www.cdc.gov>), US Department of Labor (<https://www.dol.gov>), Singapore Ministry of Health (SMH) (<https://www.moh.gov.sg>), Health Canada (Government department) (<https://www.canada.ca/en/health-canada.html>), Australian Government Department of Health (<https://www.health.gov.au>), Substance Abuse and Mental Health Services Administration (SAMHSA) (<https://www.samhsa.gov>), and Canadian Society of Addiction Medicine (CSAM) (<https://csam-smca.org>).
- Professional bodies: Royal College of Psychiatrists (RCPsych) (<https://www.rcpsych.ac.uk>), Royal College of Nursing (RCN) (<https://www.rcn.org.uk>), Royal College of Physicians (RCP) (<https://www.rcplondon.ac.uk>), American Psychiatric Association (APA) (<https://www.psychiatry.org>), Singapore Psychiatric Association (SPA) (<http://www.singaporepsychiatry.org.sg>), Singapore Medical Association (SMA) (<https://www.sma.org.sg>), Canadian Psychiatric Association (CPA) (<https://www.cpa-apc.org>), Royal Australian and New Zealand College of Psychiatrists (RANZCP) (<https://www.ranzcp.org/home>).
- Health Technology Agencies (HTA): The National Institute for Health and Care Excellence (NICE) (<https://www.nice.org.uk>), Healthcare Improvement Scotland (<https://www.healthcareimprovementscotland.org>).
- International agencies: World Health Organization (WHO) (<https://www.who.int>), Inter-Agency Standing Committee (IASC) (<https://interagencystandingcommittee.org>), UNICEF (<https://www.unicef.org>), European Monitoring Centre for Drugs and Drug Addiction (EMCDDA) (<https://www.emcdda.europa.eu/emcdda-home-page_en>).
- Scientific societies: The National Association of Psychiatric Intensive Care and Low Secure Units (NAPICU) (<https://napicu.org.uk>), RCPsych with British Geriatric Society and European Delirium Association (<https://www.bgs.org.uk>), Massachusetts General Hospital Psychiatry (<https://www.massgeneral.org/psychiatry>), World Psychiatry Association (WPA) (<https://www.wpanet.org>), British Association of Psychopharmacology (BAP) (<https://www.bap.org.uk>), Scottish Health Action on Alcohol Problems (SHAAP) (<https://www.shaap.org.uk>).
